# An origami-based colorimetric sensor for detection of hydrogen peroxide and glucose using sericin capped silver nanoparticles

**DOI:** 10.1038/s41598-023-34299-1

**Published:** 2023-05-01

**Authors:** Younes Mirzaei, Ali Gholami, Azarmidokht Sheini, Mohammad Mahdi Bordbar

**Affiliations:** 1 Department of Analytical Chemistry, Faculty of Chemistry, University of Kashan, Kashan, 87317−51167 Iran; 2 Department of Mechanical Engineering, Shohadaye Hoveizeh Campus of Technology, Shahid Chamran University of Ahvaz, Dashte Azadegan, Ahvaz, Khuzestan Iran; 3 Personal laboratory, Fasa, 74614 Iran

**Keywords:** Chemistry, Nanoscience and technology

## Abstract

The hydrogen peroxide (H_2_O_2_) measurement is considered highly important in industrial wastewater quality assessment, environmental protection, and disease detection. Here, a simple high-performance paper-based sensor is proposed for rapid and in situ detection of H_2_O_2_. To this end, 3,3′,5,5′-tetramethylbenzidine is embedded in the sensor to act as a color indicator, whose reaction with hydrogen peroxide is catalyzed by a silver nanozyme modified by sericin. The result of the reaction clarified by the appearance of blue color in the sensor detection zone is received by a portable scanner, while also calculating its intensity by image analysis software. This method is sensitive to hydrogen peroxide in the concentration range of 0.5‒240 mg/dL, providing a detection limit of 0.15 mg/dL. The ability of the sensor to determine glucose is also evaluated by adding a layer containing glucose oxidase enzyme to the sensor structure. A desirable response is obtained in the range of 1.0‒160 mg/dL, together with a detection limit of 0.37 mg/dL. Accordingly, the proposed sensor shows satisfactory results compared to clinical methods for monitoring the amount of glucose in biological samples such as serum and saliva.

## Introduction

Although hydrogen peroxide (H_2_O_2_) plays an effective role in pharmaceutical, food, mining and textile industries as a simple antimicrobial, cleaning or oxidizing compound, it has adverse effects on human health at high concentrations, and damages the skin, digestive system and respiratory tracts seriously^1^. However, acting as a chemical marker, H_2_O_2_ can directly or indirectly help diagnose cardiovascular diseases or metabolic disorders^2^. In the indirect process, it is possible to determine the amount of glucose in biological samples, followed by diagnosing diabetes by monitoring H_2_O_2_ values^3^. In a typical reaction, glucose produces gluconic acid and H_2_O_2_ in the presence of glucose oxidase enzyme^4^.

The amount of the desired product can then be evaluated by chromatography, spectroscopy, or electrochemical methods^5^. For a simpler detection mechanism, one can use a colorimetric sensor, whose response is visible to the naked eye^6^. In these methods, H_2_O_2_ can be captured with a selective color detector or can participate in an oxidation reaction of redox substrates such as 3,3′,5,5′-tetramethylbenzidine (TMB), o-phenylenediamine, and 2,2′-azino-bis(3-ethylbenzothiazoline-6-sulfonic acid) (ABTS). In the latter, peroxidase enzymes such as horseradish peroxidase catalyze the reaction^7–9^.

Despite the unique catalytic activity of peroxidase enzymes in accelerating biochemical processes, the use of these materials in the fabrication of sensing devices is limited by their price and stability under different temperature and pH conditions^10,11^. A suitable alternative is nanostructures prepared from noble metals, metal oxides, and carbon materials with catalytic behavior similar to natural enzymes^12^. These inexpensive compounds (so-called nanozymes) are easily prepared in monometallic, alloy or hybrid forms, having good stability and tunable catalytic activity by changing their shape and size^13^.

Among metal-based nanozymes, the catalytic activity of gold, silver, and platinum nanoparticles has been reported in several studies^12^. However, silver nanozymes have more commercial applications due to their unique optical properties and chemical stability, as well as their easy and cost-effective synthesis process^14^. The metal nanozymes are susceptible to foreign species in complex mixtures, which can disrupt their performance. The effect of interferences can be removed by covering the nanoparticle surface with coating agents. In addition, compared to enzymes, the coating agents increase the tendency of nanozymes to interact with the desired analyte^12,15^.

The coating agents can be polymeric compounds that are chemically synthesized or extracted from natural sources^16^. The preparation of chemical compounds requires harmful substances and special synthesis conditions, while also consuming large amounts of energy and increasing costs. In contrast, natural materials are non-toxic, biocompatible, inexpensive, and readily available^17^. The natural protein materials can be obtained from extracts of plants, flowers, tissues or microorganisms^17^. For example, sericin is a gum-like protein produced by silkworms. The structure of this protein consists of a sequence of 18 amino acids, being dominated by the contribution from serine, aspartic acid, and glycine^18,19^.

The peroxidase behavior of nanozymes in the hydrogen peroxide decomposition reaction can be realized in a liquid phase or on the surface of a polymer substrate^20,21^. In the latter, the resulting sensors need small volumes of analyte and reagents, do not use sophisticated measuring instruments, are easily portable, and show appropriate potential for point-of-care detections^22^. A suitable substrate should have the following properties: (i) high chemical and mechanical stability against hydrogen peroxide, (ii) high performance for implementing various sensor patterns on its surface, (iii) the analyte flow on its surface without the need of external force, and (iv) good safety and biocompatibility^16^. Among various substrates such as paper, glass and plastic, it has been shown that paper is suitable for fabricating the point-of-care sensors^23^.

A unique feature of paper is its flexibility, allowing for making three-dimensional (3D) origami sensors associated with the vertical transfer of the sample along multiple layers of paper^24^. Origami (or 3D) systems are recommended in the preparation of sensors based on disease diagnosis, thereby reducing and even eliminating the effect of color, turbidity and viscosity of biological samples on the response of the sensor^23^.

This study investigates the catalytic activity of silver nanozyme coated with sericin for the quantitative analysis of hydrogen peroxide. According to our knowledge, no previous report is found in the literature in this regard. The nanozyme is embedded in the substrate of a paper sensor with an origami structure. The sensor response to different amounts of exposed hydrogen peroxide is determined by tracking the color change of a redox substrate. Factors such as the type of redox substrate and its concentration, nanoparticle concentration, type, concentration and pH of buffer, temperature, and interaction time can affect the color intensity of the sensor, which must be optimized. In continuance, the efficiency of the sensor for detection of hydrogen peroxide, produced from the enzymatic reaction of glucose in the presence of glucose oxidase, is evaluated. At the end, the results of this assay for determination of glucose in the biological sample are compared with the data obtained from the standard clinical methods.

## Results and discussion

The sericin extracted from the silkworm cocoon was used as a green coating agent in the synthesis process of AgNPs. The synthesis procedure and the characterization of NPs are descried in supplemental document (Fig. S1 and Table S1). Similar to peroxidase enzymes, these NPs can catalyze the reaction of hydrogen peroxide with a redox color substrate, thus leading to a change in the color of the indicator. This capability was implemented for paper to fabricate a colorimetric sensor with rapid and in situ detection of hydrogen peroxide. Since hydrogen peroxide is the product of the glucose oxidation reaction in the presence of glucose oxidase enzyme, the performance of the fabricated sensor is verified for the detection of the concentration of glucose, as presented in detail in the following sections.

### Peroxidase mimicking properties of sericin-AgNPs

**Figure 1:**
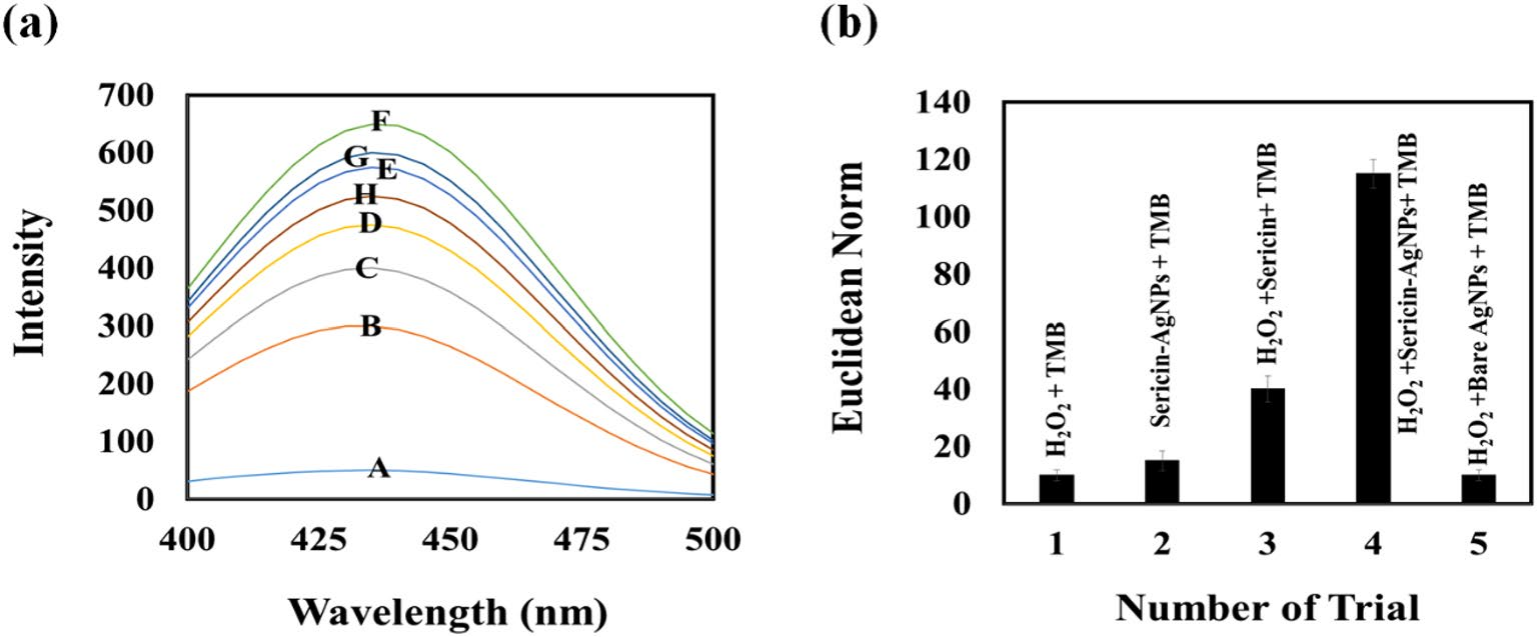
(**a**) Evaluation the peroxidase activity of sericin-AgNPs by monitoring the fluorescence emission of 2-hydroxy terephthalic acid, the product of terephthalic acid and ·OH radicals interaction. The fluorescence intensity was proportional to concentration of sericin-AgNPs which was changed from 0.0 (A) to 0.1 (F) μg/ mL. (**b**) Evaluation the effect of pure H_2_O_2_, sericin-AgNPs, a mixture of H_2_O_2_ and sericin, a mixture of H_2_O_2_ and sericin-AgNPs, and a mixture of H_2_O_2_ and bare AgNPs on the TMB oxidation reaction. The analysis was done at optimum conditions.

The method proposed by Hashimoto was used to confirm the peroxidation behavior of sericin-AgNPs^25^. In a typical experiment, a probe sensitive to ·OH radicals such as 5.0 × 10^–4^ M terephthalic acid, 1.0 × 10^–2^ M hydrogen peroxide and sericin-AgNPs solution with a certain concentration in the range of 0.0–0.1 μg/ ml was mixed in 0.1 M acetic acid-sodium acetate buffer (pH = 4). The prepared mixture was heated at 40 °C for 40 min. The interaction of terephthalic acid with ·OH radicals leads to the formation of 2-hydroxy terephthalic acid. The resulting compound has fluorescence emission in the range from 400 to 500 nm. Based on the fluorescence spectra shown in Fig. 1a, the fluorescence intensity of 2-hydroxy terephthalic acid is gradually enhanced by increasing the NP concentration. The definitive reason for this enhancement is the production of ·OH radicals from the decomposition of hydrogen peroxide. This reaction is catalyzed by sericin-AgNPs due to their pseudo-peroxidation properties.

In the colorimetric detection of hydrogen peroxide, the analyte decomposes after being bound to the surface of AgNPs, thereby producing ·OH radicals. These radicals are stabilized on the surface of the NPs due to partial electron exchange interaction. The NPs can be adsorbed to the surface of the redox indicator through electrostatic interaction. As a result, the conditions for the oxidation of the indicator with •OH radicals are provided, which can in turn form a colored product^26^.

As a proof of concept, five different tests were performed on the paper surface under optimal conditions (described in the next section), in which TMB was exposed to pure H_2_O_2_, sericin-AgNPs, a mixture of H_2_O_2_ and sericin, a mixture of H_2_O_2_ and sericin-AgNPs, and a mixture of H_2_O_2_ and bare AgNPs. As shown in Fig. 1b, the formation of oxidized TMB along with the appearance of intense blue color occurs only in the simultaneous presence of H_2_O_2_ and sericin-AgNPs, indicating the catalytic role of sericin-AgNPs in the decomposition of H_2_O_2_ and the interaction of the radical product with the redox indicator.

### Optimal conditions for determination of hydrogen peroxide

The response of the paper sensor for optimal measurement of hydrogen peroxide significantly depends on the catalytic activity of nanozyme in the oxidation reaction of the redox indicator. Therefore, the efficiency of the fabricated sensor can be increased by optimizing factors such as the type and concentration of the redox indicator, NP concentration, and pH and concentration of buffer. Also, temperature and time of interaction should be optimized. To perform these experiments, 5.0 μL of hydrogen peroxide solution with a concentration of 140.0 mg/dL was used.

For the initial test, NP solution (0.5 mg/ml) was injected into the nanozyme zone. The detection zone was filled with a mixture of redox indicator and acetic acid-sodium acetate buffer with a concentration of 0.05 mol/L (pH = 4). The final concentration of indicator (TMB, OPD or ABTS) was equal to 5.0 × 10^–3^ mol/L. By adding H_2_O_2_ to the injection zone, the color change of the indicators was from colorless to blue, yellow, and green for TMB, OPD, and ABTS, respectively (Fig. S2a). As observed, the synthesized nanozyme shows better peroxidase behavior for the oxidation of TMB.

By changing the concentration of TMB in the range of 1.0 × 10^–3^ mol/L – 9.0 × 10^–3^ mol/L, the response of the sensor gradually increased up to 5.0 × 10^–3^ mol/L. No significant change was observed in the color of the indicator at higher concentrations (Fig. S2b).

To optimize the concentration of nanozyme, different solutions were prepared with concentrations in the range of 0.1–1.3 mg/ml. The catalytic behavior of the nanozyme is fixed at 0.5 mg/ml (Fig. S2c). To continue the study, the concentration of nanozyme was chosen to be 0.5 mg/ml.

The catalytic activity of nanozymes depends on the pH of the medium. For example, the decomposition of hydrogen peroxide in the presence of silver nanozymes leads to the production of ·OH radicals at acidic pH or release of O_2_ at alkaline pH^27^. Since ·OH radicals are needed to oxidize TMB, it is preferable to follow the reaction in an acidic medium. The catalytic activity of nanozyme was investigated using acetic acid-sodium acetate buffer with a concentration of 5.0 × 10^–2^ mol/L in a pH range of 3.0‒7.0. As seen in Fig. S2d, the catalytic behavior of nanozyme, and consequently the response of the sensor can be improved by changing the pH of medium from 3.0 to 4.5. In the case of more acidic media, the high concentration of hydronium ions prevents ·OH radicals or TMB from adsorption on the surface of the nanozyme, thereby avoiding the occurrence of the redox oxidation reaction. At higher pH values, the solubility of TMB in the buffer decreases, resulting in a negative effect on the sensor response^28^.

After adjustment of the pH value, the experiment was continued by varying the buffer concentration in the range from 0.01 mol/L to 0.15 mol/L. According to Fig. S2e, the best response of the sensor is observed in the buffer medium with a concentration of 0.05 mol/L.

In order to investigate the effect of temperature on the peroxidase activity of nanozyme, the reaction of hydrogen peroxide with TMB was repeated in the temperature range of 25‒55 °C with 5 °C steps. As seen in Fig. S2f, the oxidation process of TMB and the formation of blue diamine/diimine complex are enhanced by increasing the temperature from 25 °C to 35 °C^28^. At higher temperatures, the efficiency of the sensor decreases due to some reasons such as the destruction of the silver nanozyme, the inactivation of the nanozyme activity, or the formation of a yellow diamine product^28^.

To determine the time required for the complete interaction of hydrogen peroxide with TMB, the study was followed at two temperatures (25 °C and 35 °C). It was found that the color change of the sensor at temperatures of 35 °C and 25 °C was constant at the same value after 3.0 (Fig. S2g) and 4.5 (Fig. S2h) min, respectively. Since the purpose of the study was to develop a user-friendly device for detection of hydrogen peroxide and glucose, and due to the fact that providing heating devices was not possible in all conditions, the experiment was preferred to take place at a temperature of 25 °C and the data was collected after 4.5 min.

### Quantitative analysis of hydrogen peroxide

**Figure 2:**
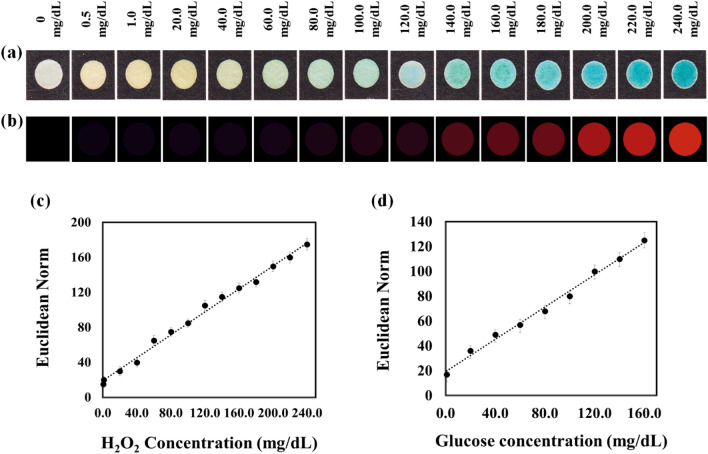
(**a**) Variation the color of redox indicator in the presence of H_2_O_2_ (0.5–240.0 mg/dL) and glucose (1.0–160.0 mg/dL). (**b**) The respective color difference map. (**c**) and (**d**) The calibration plots for determination of H_2_O_2_ and glucose, respectively. The analysis was done at optimum conditions.

After developing the sensor with optimized components, the color changes of TMB were evaluated in the presence of different hydrogen peroxide concentrations (0.0‒300.0 mg/dL). From Fig. 2a, the color of the sensor changes from colorless to intense blue when increasing the hydrogen peroxide concentration The variation in sensor response is shown more noticeably in the color difference map (Fig. 2b). According to Fig. 2c, the response of the sensor has a linear relationship with the concentration of hydrogen peroxide in the range of 0.5‒240.0 mg/dL. For this assay, the detection limit is obtained to be 0.15 mg/dL. The analytical characteristics of the proposed sensor are summarized in Table 1.

**Table 1:** The features of two paper based sensors for determination of H_2_O_2_ and glucose.

Parameter	Value	
	H_2_O_2_	Glucose
Linear range (mg/dL)	0.5–240.0	1.0–160.0
Detection limit (mg/dL)	0.15	0.37
R^2^	0.994	0.992
Linear equation	6.5 C_H2O2_ + 19.28	6.1 C_glucose_ + 19.16
Calibration sensitivity	6.5	6.1
Analytical sensitivity	0.8 (For 220.0 mg/dl)	0.9 (For 160.0 mg/dl)
RSD (%) (for 5 measurements)	5.07 (For 220.0 mg/dl)	5.36 (For 160.0 mg/dl)

In order to assess the reproducibility of the assay, five individual sensors were exposed to 220.0 mg/dL of the analyte, followed by calculating the relative standard deviation of the determinations after receiving the results. The error rate of 5.07% is indicative of the good reproducibility of the colorimetric analysis of hydrogen peroxide.

### Optimal conditions for glucose detection

The difference between the proposed assay devices for H_2_O_2_ and glucose detection was only in one layer (i.e. the GOx enzyme zone). Therefore, the components of this layer were optimized for glucose detection, and the results are described in the following text. The enzyme zone includes GOx enzyme and an immobilizer. The immobilizing agent causes the enzyme to be physically attached to the surface of paper, thus avoiding its leakage from the zone^29^. Generally, BSA is employed as an immobilizer to prevent the GOx leakage^29^. In practical analysis, the enzyme zone was modified by BSA (10.0 mg/mL), followed by adding GOx with the same concentration to this layer. 10.0 μL of glucose solution (120.0 mg/dL) was injected into the sensor, and TMB color changes were monitored.

In the first step of optimization, the molar ratio of BSA to GOx was optimized. According to Fig. S3a, the sensor response increases by increasing the ratio from 0.3 to 0.5. For higher ratios, a downward trend is observed in the Euclidean norm due to a decrease in the enzyme activity at high concentrations of BSA. In the next step, the enzyme concentration was changed from 5.0 to 25 mg/mL. The bar graphs in Fig. S3b show a maximum value for a concentration of 15.0 mg/mL. No significant change is seen in the color of the TMB, and consequently in the response of the sensor at higher concentrations. To achieve the optimal response time, color changes of the sensor were investigated for a time interval between 0.0 and 10 min. The graph presented in Fig. S3c reveals that the time required for the detection of glucose is about 7 min.

### Glucose determination

Different concentrations (0.0‒240.0 mg/dL) of glucose solution were injected into the prepared device. Based on the calibration plot (Fig. 2d) and the statistical information (Table 1), a good linear relationship is established between the assay responses and glucose concentrations in the range of 1.0‒160.0 mg/dL. In this case, the limit of detection is obtained to be 0.37 mg/dL. Also, the responses of sensor were collected for five individual analyses of glucose with a concentration of 160.0 mg/dL. The error value of about 5.36% confirms the reproducibility of the determination process using the proposed method.

### Evaluation of stability

To evaluate the stability of the glucose sensor, several paper devices were prepared under the same conditions. The provided sensor was stored in a plastic bag and transferred to a refrigerator at a temperature of 4 °C. Each sensor was exposed to a glucose solution with a concentration of 120.0 mg/dL at a time period of 7 days. This process took 56 days. Figure S4 indicates that the response of the method to a certain amount of glucose remains almost constant for 42 days, and after that it decreases significantly. This can be due to a reduction in the activity of the GOx and nanozyme or due to the oxidation of the TMB during physical and chemical changes. Table S2 illustrates the comparison between the amounts of the Euclidean norm of the sensor in the presence of glucose (120.0 mg/dL) at three different times, including immediately after the fabrication, and after 42 and 49 days. Taking into account the values obtained for the t-test and the relative error, the color changes of the sensor are found to be proportional to the glucose concentration only up to 42 days after the sensor fabrication.

### The effect of foreign species

**Figure 3:**
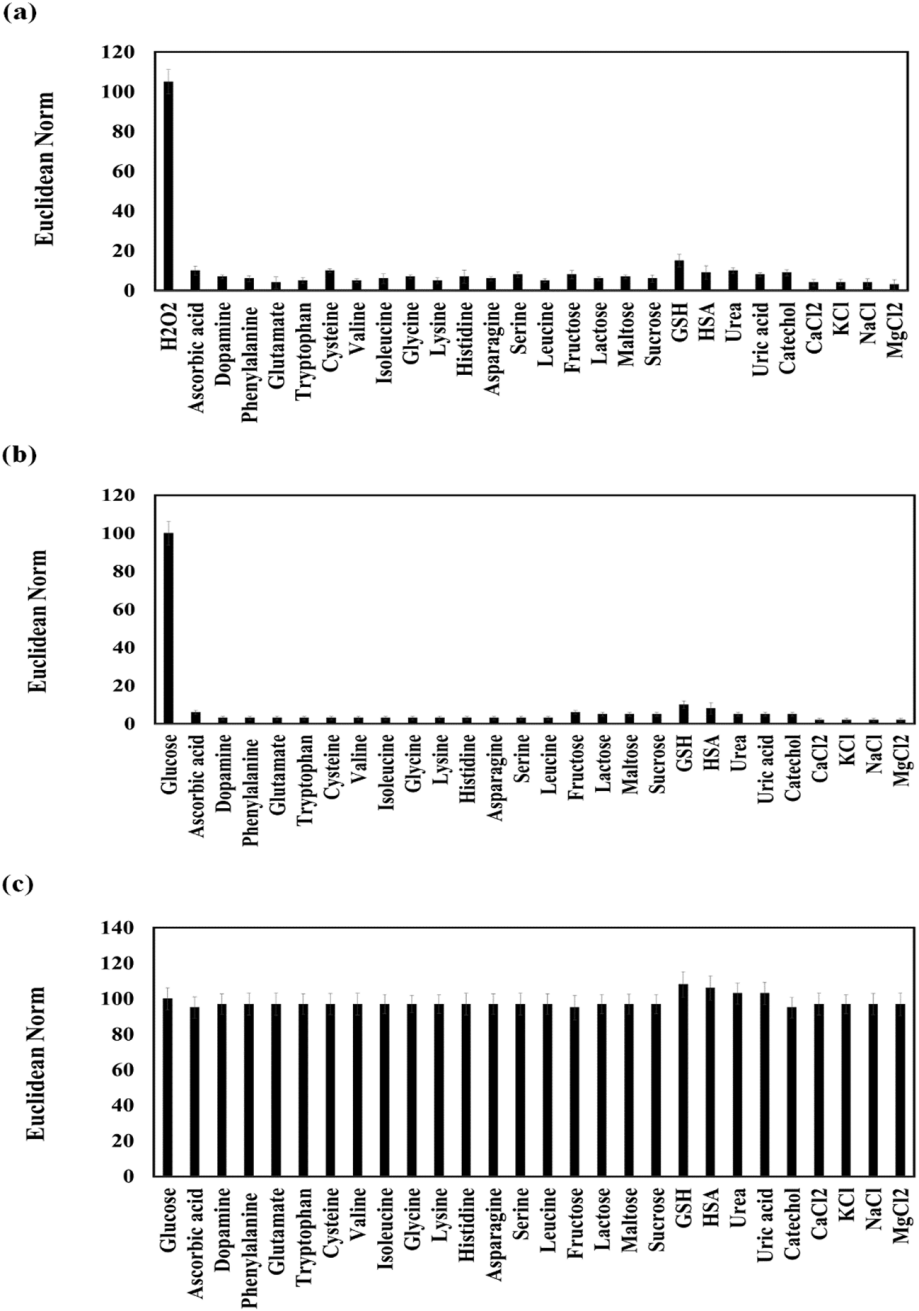
The response of sensor in the presence of H_2_O_2_ (**a**) and glucose (**b**) and foreign species, individually. (**c**) Evaluation of the sensor ability for determination of glucose alone or after mixing by a specified foreign species. In each mixture, the concentration of interferences were 20 times higher than the glucose concentration. The analysis was done at optimum conditions.

To evidence high-selectivity of the proposed sensor to H_2_O_2_ and glucose, These analytes and other effective foreign species such as ascorbic acid, dopamine, phenylalanine, glutamate, tryptophan, cysteine, valine, isoleucine, glycine, lysine, histidine, asparagine, leucine, fructose, lactose, maltose, sucrose, glutathione (GSH), human serum albumin (HSA), urea, uric acid, catechol, calcium chloride (CaCl_2_), potassium chloride (KCl), sodium chloride (NaCl), and magnesium chloride (MgCl_2_) were injected separately into the injection zone of the sensor. The concentration of each species was adjusted to 120.0 mg/dL. As can be seen in Fig. 3a and b, a significant response is recorded for H_2_O_2_ and glucose, and the color changes are not striking for other species.

In a parallel experiment, the sensor was exposed to pure glucose, and a mixture of glucose and a foreign species. The concentration of glucose was 120.0 mg/dL, and the amount of other species was considered to be 20 times higher than the analyte concentration. From Fig. 3c, no significant difference is found between the responses of the sensor for the pure glucose and the mixture of glucose and foreign species.

### Detection of glucose in biological samples

The validity of the measurement assay was evaluated by determining the concentration of glucose in biological samples. For this purpose, saliva and serum samples were collected from a specific individual. After the pre-preparation, each biological sample was divided into five individual tubes with an equal volume. Next, different concentrations of glucose solution with an equal volume were added to each tube. One tube did not contain the standard glucose solution. Moreover, each sample was diluted 10 times. 10.0 µL of saliva or serum sample was injected into the proposed sensor. The sensor responses were recorded after 7 min. On the other hand, the amount of glucose in the prepared samples was calculated by a standard clinical method. The results of both the proposed and standard methods are given in Table 2. The information from the *t* test confirms a good relationship between the data of both methods. The low value of the relative error indicates that the proposed method is highly accurate.

**Table 2:** Determination of glucose in saliva and serum samples using proposed and clinical methods.

Sample	Number of sample	Found^1^ (mg/dL)		t_experimental_^2^	Relative error (%)	Recovery^3^ (%)
		Proposed method	Clinical method			
Saliva	1 (0.0 mg/dL)	1.3 (± 0.044)	1.25 (± 0.040)	1.88	4.0	–
	2 (2.0 mg/dL)	3.5 (± 0.13)	3.39 (± 0.071)	1.67	3.2	110.0
	3 (4.0 mg/dL)	5.6 (± 0.18)	5.42 (± 0.15)	1.71	3.3	107.5
	4 (10.0 mg/dL)	11.7 (± 0.48)	11.40 (± 0.32)	1.16	2.6	104.0
	5 (16.0 mg/dL)	18.0 (± 0.75)	17.51 (± 0.61)	1.33	2.8	104.4
Serum	1 (0.0 mg/dL)	75.4 (± 2.75)	73.13(± 2.16)	1.45	3.1	–
	2 (25.0 mg/dL)	104.1 (± 4.61)	101.10(± 3.63)	1.14	3.0	114.8
	3 (40.0 mg/dL)	122.3 (± 5.32)	115.56(± 4.27)	2.21	5.8	117.2
	4 (50.0 mg/dL)	129.2 (± 4.49)	124.36(± 4.24)	1.75	3.9	107.6
	5 (65.0 mg/dL)	147.4 (± 5.85)	140.89(± 4.08)	2.04	4.6	110.7

^1^Mean of 5 measurements.

^2^t_critical (8, 0.05) = 2.31.

^3^Recovery is the ratio of the value found by the proposed sensor to the added value.

In recent years, the several colorimetric-based sensors have been proposed, employing peroxidase properties of nanostructures to detect glucose. The catalytic activity of these nanostructures can affect the sensitivity and selectivity of the sensor response for determining a specific analyte. The performance of some of these sensors is presented in Table S3. As can be seen, the detection limits of sensors made in the solution are better than those of paper sensors. Probably, the catalytic behavior of the nanostructure is limited after being immobilized on the surface of paper. However, sensors fabricated using paper substrates can be made with simple and lower price design and determine glucose in an extensive concentration range. It is clear that the best linear range is obtained for sericin-AgNPs. Of course, compared to solution-based sensors, the proposed sensor is more user-friendly because it is portable, needs a lower volume of sample and reagents, and does not require unsafe glassware and costly devices to achieve a colorimetric response. Also, unlike other paper-based sensors, the method proposed here is realized by an origami structure, resulting in the transfer of analyte from the injection zone to the detection zone in a shorter time. Moreover, the analysis time is reduced, and interfering agents of the biological sample are eliminated.

## Conclusions

Two paper-based colorimetric sensors have been developed for determination of hydrogen peroxide and glucose using the peroxidase properties of sericin-capped AgNPs, and the flexibility of the paper substrate. The synthesized nanozyme was a green compound with a simple, low-cost and safe synthesis process, allowing for catalyzing the oxidation reaction of various redox substrates. The high catalytic activity of the proposed nanozyme led to the decomposition of small amounts of hydrogen peroxide, providing sensors with acceptable sensitivity. The responses of the fabricated sensors were linear in a wide concentration range of hydrogen peroxide and glucose, enabling the identification of analytes in various biological, environmental and food samples. The resulting sensors with high selectivity remained stable for 6 weeks against environmental changes. Considering its appropriate performance for detection of glucose in the biological samples, the paper sensor can be introduced as a reliable alternative for fast and non-invasive glucose measurement.

## Method

### Materials

Hydrogen peroxide (H_2_O_2_), acetic acid (CH_3_COOH), silver nitrate (AgNO_3_), sodium hydroxide (NaOH), and hydrochloric acid (HCl) were purchased from Merck Company. Glucose, ascorbic acid, dopamine, phenylalanine, glutamate, tryptophan, cysteine, valine, isoleucine, glycine, lysine, histidine, asparagine, serine, leucine, fructose, lactose, maltose, sucrose, urea, uric acid, catechol, calcium chloride (CaCl_2_), potassium chloride (KCl), sodium chloride (NaCl), terephthalic acid, magnesium chloride (MgCl_2_), sodium acetate (NaCH_3_COO), glucose oxidase from Aspergillus niger, TMB, o-phenylenediamine (OPD), and ABTS, human serum albumin (HSA), glutathione (GSH) were provided by Sigma Aldrich Company. These materials were of analytical grade with high purity. Bombyx mori silk cocoons were collected from Tehran local market. The paper substrate was created by Whatman Grade NO. 2 Filter Paper. The procedures for extraction of sericin and synthesis of nanozyme were explained in supporting information document.

### Instruments

UV–Vis (OPTIZEN 3220UV, South Korea) and FT-IR (Perkin-Elmer 781) spectrophotometers were used to capture UV–Vis and IR spectra of synthesized nanoparticles (NPs), respectively. Zetasizer Nano ZS90 (Malvern, UK) was employed to determine both hydrodynamic size and surface electrical charge of the prepared NPs. A flatbed scanner (CanoScan LiDE 700F, USA) with a resolution of 300 dpi was used to receive desired images. The images were investigated by ImageJ software (1.52a, National Institutes of Health, USA, https://imagej.nih.gov/ij). The sensor pattern was designed by AutoCAD 2016 software, and printed on the surface of paper using HP LaserJet printer 1200.

### Design of sensor pattern

**Figure 4:**
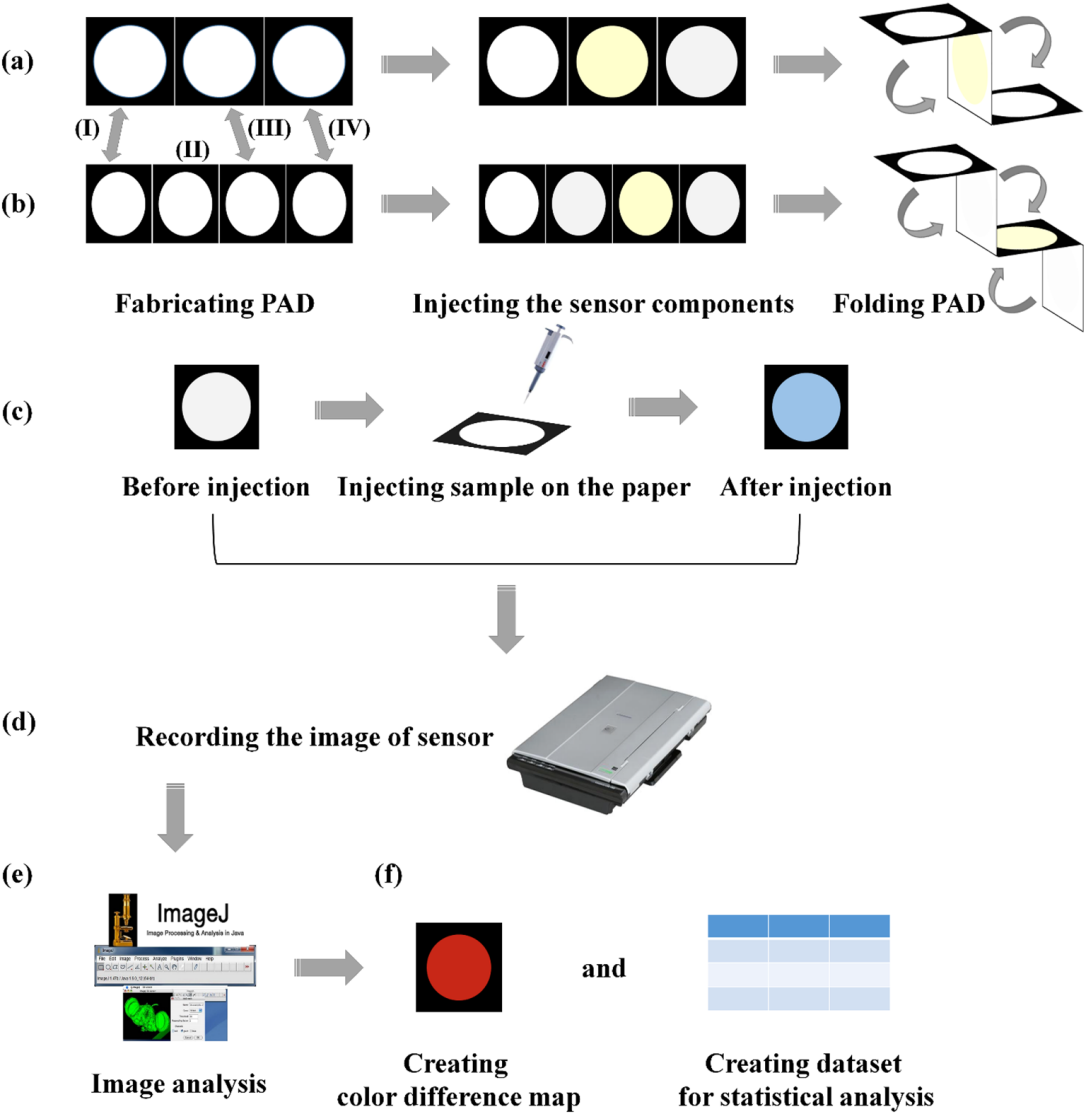
A summary of the experimental process: (**a**) and (**b**) sensor fabrication process for detection of H_2_O_2_ and glucose, respectively. In these parts, (I), (II), (III) and (IV) are defined as injection, enzyme, nanozyme and detection zones, respectively. (**c**) The injection process of sample, followed by changing in the color of redox indicator. (**d**) Recording the image of sensor before and after exposing to analyte. (**e**) Image analysis process, (**f**) indicating the response of sensor by color difference map and collecting them in a dataset for statistical analysis.

The purpose of this study was the determination of hydrogen peroxide, and subsequently the detection of glucose. Therefore, two paper substrates with the same shape were designed. For the first study, the substrate had dimensions of 1 cm × 3 cm, and included three layers defined as the injection, nanozyme, and detection zones. Each zone was composed of black and white parts, representing hydrophobic and hydrophilic areas, respectively. For glucose detection, a zone consisting of glucose oxidase (GOx) enzyme was inserted between the injection and nanozyme zones. These designs were performed by AutoCAD software. A printer was used to print the patterns on the paper surface. The paper was then transferred to an oven and heated at 200 °C. This process took 45 min, resulting in the penetration of the ink into the texture of the paper and increasing the hydrophobicity of the black areas^30^. The designed patterns are presented in Fig. 4.

### Sensing procedure

In this part, 1.0 μL of the sensor components were injected in their respective zones: sericin-AgNPs was poured in the nanozyme zone while the detection zone was filled with a mixture containing redox detector and acetic acid-sodium acetate buffer. As mentioned in the previous section, the sensor made for detecting glucose has an additional for immobilization of the GOx enzyme. It should be noted that an immobilizer agent such as BSA was needed to place the enzyme on the substrate. The immobilizing agent was used to prevent the enzyme from leakage due to the passage of the analyte^31^. The paper was folded according to the design shown in Fig. 4. It is possible to use a holder for keeping all the layers completely overlapped and connected with each other.

A certain volume and concentration of the sample (containing hydrogen peroxide or glucose) was added to the injection area (Fig. 4c). The solutions with concentration ranges of 0.0‒300.0 mg/dL and 0.0‒240.0 mg/dL were prepared and exposed to the sensor for the quantitative measurement of hydrogen peroxide and glucose, respectively. The color change resulting from the oxidation process of the redox indicator in the presence of hydrogen peroxide (in pure form or produced from the enzymatic reaction of glucose) and the synthesized nanozyme can be seen with the naked eye. These changes can also be recorded using a scanner (Fig. 4d). The photos before and after the sample injection were analyzed with ImageJ software, providing three average values for red, green and blue color elements of each photo. For each experiment, the color element values of the photos taken before and after the injection were subtracted from each other (Fig. 4e). The difference values were collected in a ternary vector and used to prepare the color difference map (Fig. 4f). The Euclidean norm of the vector was also calculated, representing the final response of the sensor as follows:

1 $$Euclidean\,\, norm=\sqrt{{(\Delta R)}^{2}+{(\Delta G)}^{2}+{(\Delta B)}^{2}}$$

where ∆ is the difference in the values of R, G and B color elements for the detection zone of the sensor before and after the interaction. Note that, each experimental analysis was performed for 3 times.

### Real sample evaluation

The colorimetric sensor designed in this study was employed to detect glucose in saliva and serum samples. To this end, saliva and serum samples of one of the authors (a healthy person) were collected in sterile tubes. Note that, before the test, the saliva sample was centrifuged at 5000 rpm for 10 min. The serum sample was also mixed with trichloroacetic acid (10%) and centrifuged at 3000 rpm for 10 min. Thereby, physical particles were removed from the saliva sample, and proteins and amino acids sedimented in the serum sample^31^. The prepared solutions were mixed with certain concentrations of glucose. The optimal volume of each prepared sample was added to the paper sensor. On the other hand, all samples were analyzed by a clinical detection method. Finally, the comparison between the data obtained from the proposed and clinical methods was evaluated using the *t* test statistical analysis.

## Supplementary Information

Supplementary Information.

## Data Availability

The datasets used and/or analyzed during the current study are available from the corresponding author on reasonable request.
