# Supplementary material for: An origami-based colorimetric sensor for detection of hydrogen peroxide and glucose using sericin capped silver nanoparticles
Source: Sci Rep. 2023 May 1;13:7064. doi: 10.1038/s41598-023-34299-1 (PMC10151347; doi:10.1038/s41598-023-34299-1)
Supplement: Supplementary file 1 — Supplementary Information. [file 41598_2023_34299_MOESM1_ESM.docx]

Supporting information

for

**An origami-based colorimetric sensor for detection of hydrogen peroxide and glucose using sericin capped silver nanoparticles**

Younes Mirzaei^1^, Ali Gholami^1,*^, Azarmidokht Sheini^2^, Mohammad Mahdi Bordbar^3^

^1^Department of Analytical Chemistry, Faculty of Chemistry, University of Kashan, Kashan, 87317−51167, Iran.

^2^Department of Mechanical Engineering, Shohadaye Hoveizeh Campus of Technology, Shahid Chamran University of Ahvaz, Dashte Azadegan, Khuzestan, Iran.

^3^Independent researcher, Personal laboratory, Fasa, 74614, Iran.

***** Email address: agholami@kashanu.ac.ir

**Addition information**

1. Sericin extraction

Bombyx mori silk cocoons were rinsed twice with deionized water and allowed to dry in free air at room temperature for 2 days. The dried cocoons were then cut into pieces. It should be note that the cocoons did not contain any moisture. The crushed pieces were put into a container of boiling deionized water. The extraction process was continued under vigorous stirring at 50 °C . In this condition, the silk fibroin did not dissolve in water due to its hydrophobicity, and was easily separated from the solution. The sericin aqueous solution was centrifuged to remove impurities such as silk seeds. The pure solution was dried and powdered by a freeze-dryer ^1^. The resulting sericin powder was kept for further study.

2. Synthesis of sericin-capped silver nanoparticles (sericin-AgNPs)

Modified silver NPs (AgNPs) were synthesized based on previous reports ^2^. First, the solutions of 1.0 × 10^-2^ mol/L of AgNO_3_ and 1% (W/V) of sericin were prepared. 10.0 ml of AgNO_3_ solution was added to a sterile container, and was then mixed with an equal volume of sericin solution under stirring conditions. After 5 min, the reaction medium was alkalized using NaOH solution (5 M), and the pH of the mixture was adjusted to 11. The appearance of a yellow-brown color indicated the formation of AgNPs. In order to remove unreacted sericin, the NP solution was centrifuged, and the remaining precipitate was kept in a sterile tube at 4 °C.

3. Characteristics of sericin-AgNPs

The absorption spectrum of sericin-AgNPs synthesized in the form a yellow-brown compound is shown in Fig. S1a. As can be seen, a peak appears at the wavelength of 420 nm for the NPs. Based on the results of DLS method (Fig. S1b), the hydrodynamic size of the synthesized NPs is calculated to be 55 nm, and the electric charge distributed on their surface is reported to be ‒14 mV, as presented in Fig. S1c. The FT-IR spectra of pure sericin and synthesized NPs are shown in Fig. S1d. The characteristic absorption bands can be observed at 3271, 1634, 1511, 1236 and 1049 cm^-1^, corresponding to OH stretching, C=O bending, N-H bending, C-N stretching and C-H bending vibrations, respectively. These functional groups are found in the phenolic and amide structures of protein ^1^. After coating the surface of AgNPs with sericin, the intensity of the absorption peaks decreases and their position changes towards higher wavenumbers, indicating the modification of the surface with the protein.

The prepared NPs were placed in the nanozyme zone of the paper-based sensor, making hydrogen peroxide oxidize the redox indicator and change its color. To achieve a reliable colorimetric response, the immobilization process of NPs on the surface of paper needs to be reproducible. To carry out this experiment, 1.0 μL of the synthesized NPs was dripped on the surface of five individual nanozyme zones. The values of R, G and B color elements were calculated for each sensor using the image analysis software. Table S1 shows the error rate in terms of relative standard deviation for the five measurements. The low error values confirm that the nanozyme zone is filled during the reproducible process.


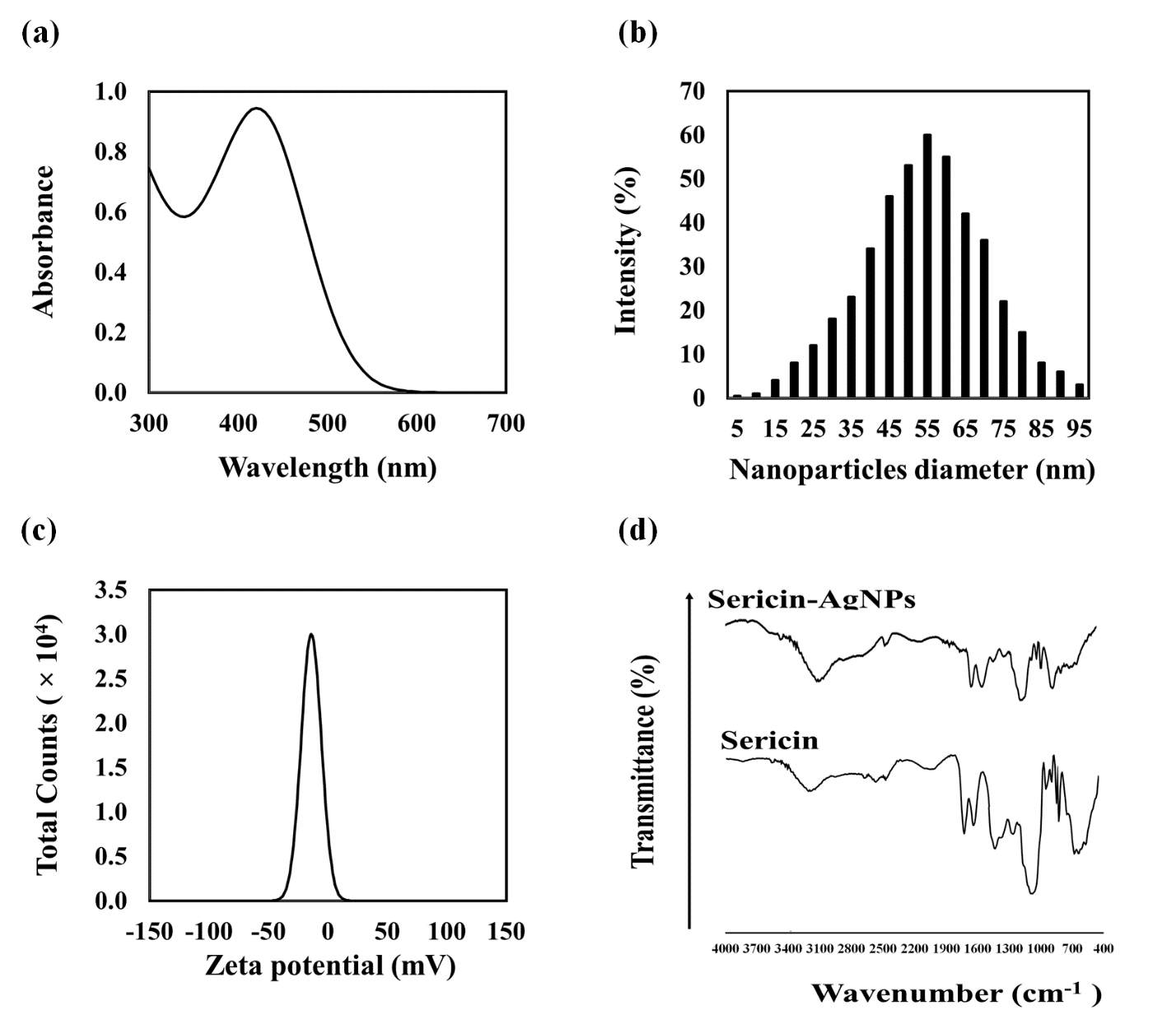


**Fig. S1.** Characterization of sericin-AgNPs. (a) UV-Vis spectrum, (b) hydrodynamic size obtained by DLS method, (c) electric charge distribution, (d) FT-IR spectrum.


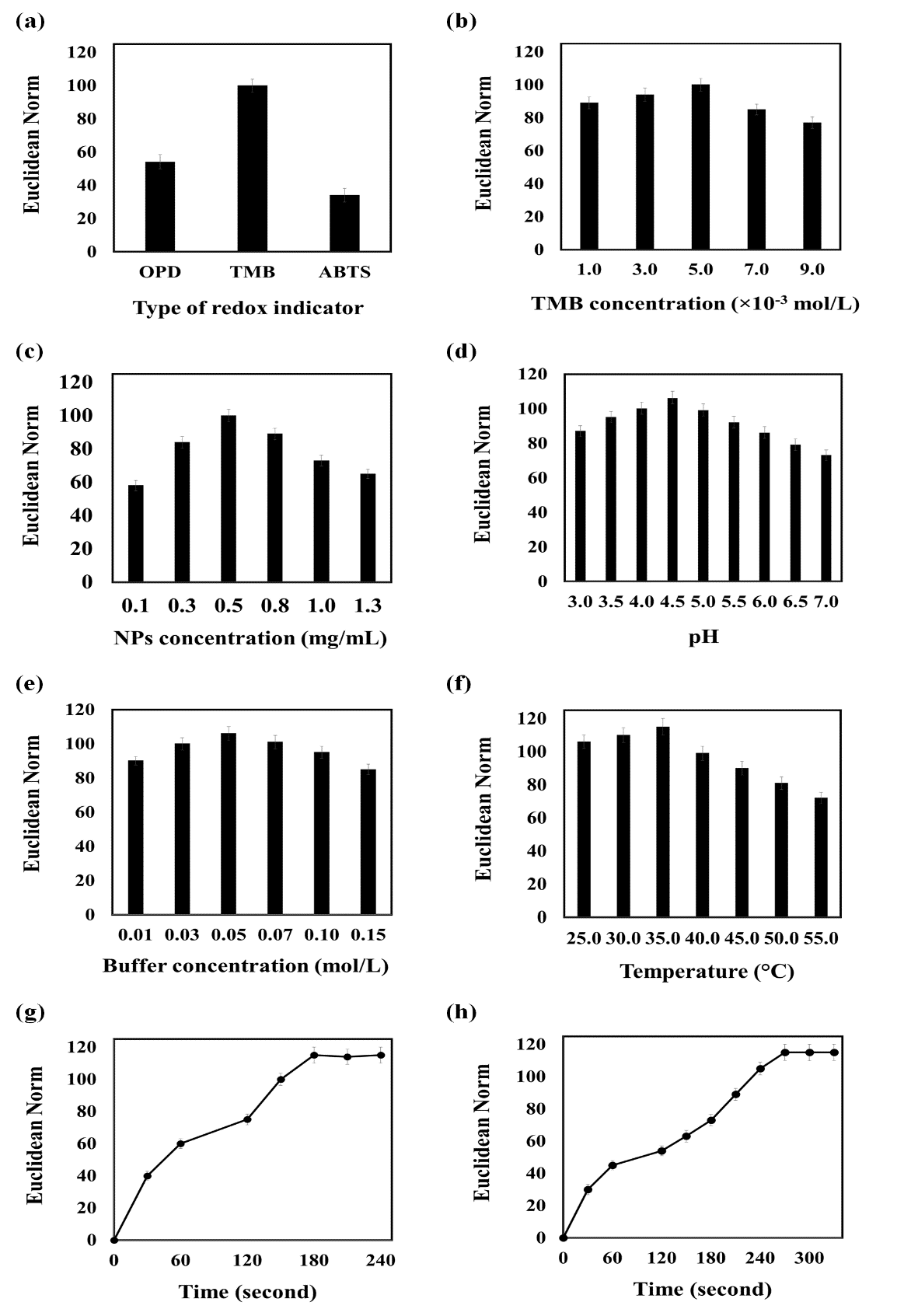


**Fig. S2.** Optimum conditions for determination of H_2_O_2_ (a) Type of redox indicator, (b) concentration of redox indicator, (c) concentration of nanozyme, (d) pH, (e) concentration of buffer, (f) incubation temperature, (g) incubation time at 35°C, (h) incubation time at 25°C.


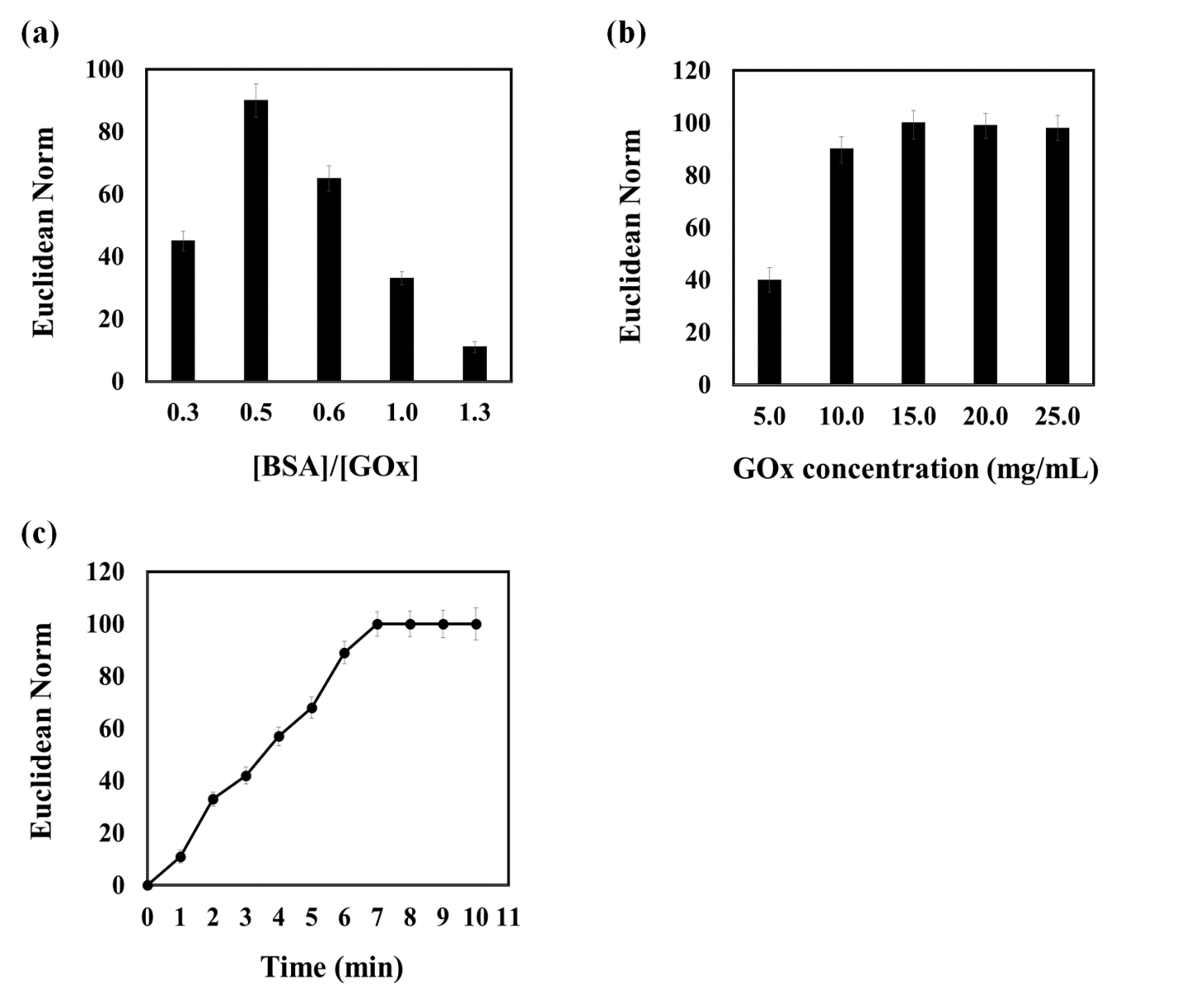


**Fig. S3.** Optimum conditions for determination of glucose (a) The molar ratio of BSA to GOx, (b) concentration of GOx, (c) The reaction time.


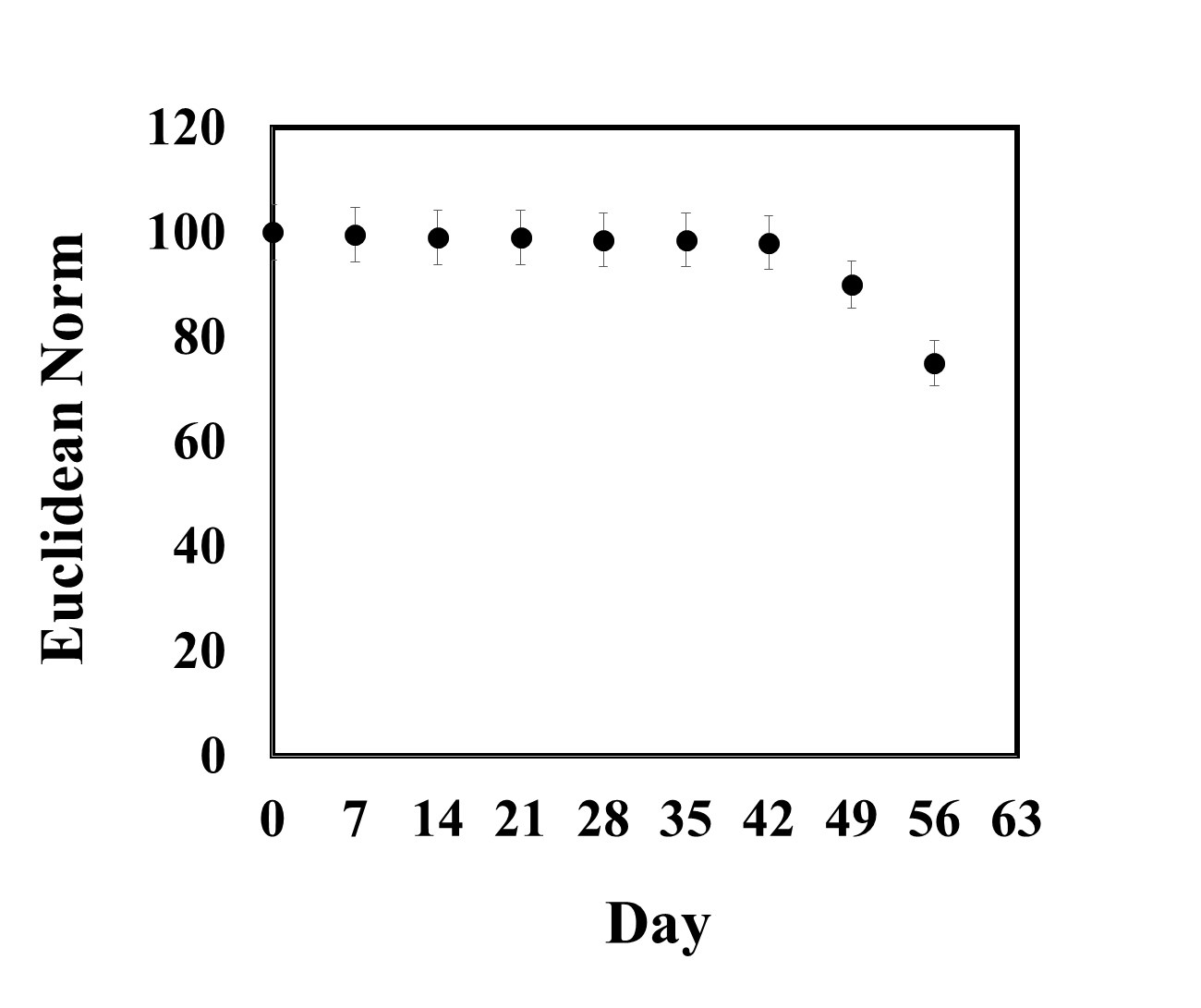


**Fig. S4.** Evaluation of the sensor stability. The analysis was done at optimum conditions.

**Table S1.** The repeatability of nanozyme zone fabrication process.

| Type of NPs | RGB values | Number of sensor | | | | | RSD % |
| --- | --- | --- | --- | --- | --- | --- | --- |
|  |  | S1 | S2 | S3 | S4 | S5 |  |
| Sericin-AgNPs | R | 225 | 227 | 223 | 225 | 224 | 0.66 |
|  | G | 204 | 202 | 207 | 204 | 201 | 1.13 |
|  | B | 150 | 153 | 149 | 150 | 151 | 1.01 |

**Table S2.** Comparison between the responses of assay for determination of glucose (120.0 mg/dL) obtaining immediately, 42 days and 49 days after sensor fabrication.

| **Euclidean norm^1^** | **Immediately**  **after sensor fabrication** | **42 days**  **after sensor fabrication** | **49 days**  **after sensor fabrication** |
| --- | --- | --- | --- |
|  | 100 (± 5.25) | 98 (± 5.18) | 90 (± 4.51) |
| **t__experimental_^2^** | - | 0.61 | 3.23 |
| **Relative error (%)** | - | -2 | -10 |
| ^1^ Mean of 5 measurements (±SD)  ^2^ t_ _critical_ (8,0.05) = 2.31 | | | |

.

| **Table S3.** Analytical characteristics of colorimetric glucose sensors fabricating by different nanozymes. | | | | |
| --- | --- | --- | --- | --- |
| **Type of Nanozyme** | **Sensor media** | **Linear range (mg/dL)** | **Detection limit (mg/dL)** | **Ref.** |
| PtS_2_ nanosheets | Solution | 0.009-2.7 | 0.004 | ^3^ |
| C-dots / V_2_O_5_ nanowires | Solution | 0.013-5.4 | 0.013 | ^4^ |
| RhNPs | Solution | 0.09-2.2 | 0.014 | ^5^ |
| Cu-Pd/rGO nanocomposites | Solution | 0.009-0.9 | 0.005 | ^6^ |
| Cu-Pd/rGO nanocomposites | Paper | 0.018-1.8 | 0.018 | ^6^ |
| Fe-MIL-101 | Paper | 0.018-2.7 | 0.045 | ^7^ |
| β-CD-Pd@Au | Paper | 7.2-90.1 | 0.47 | ^8^ |
| Sericin-AgNPs | Paper | 1.0-160.0 | 0.7 | This work |
|  | | | | |

**References:**

1. Harisha, K. S. *et al.* Eco-synthesis of gold nanoparticles by Sericin derived from Bombyx mori silk and catalytic study on degradation of methylene blue. *Part. Sci. Technol.* **39**, 131–140 (2021).

2. Gün Gök, Z., Günay, K., Arslan, M., Yiğitoğlu, M. & Vargel, İ. Coating of modified poly(ethylene terephthalate) fibers with sericin-capped silver nanoparticles for antimicrobial application. *Polym. Bull.* **77**, 1649–1665 (2020).

3. Zhang, W. *et al.* PtS2 nanosheets as a peroxidase-mimicking nanozyme for colorimetric determination of hydrogen peroxide and glucose. *Microchim. Acta* **188**, (2021).

4. Honarasa, F., Kamshoori, F. H., Fathi, S. & Motamedifar, Z. Carbon dots on V 2 O 5 nanowires are a viable peroxidase mimic for colorimetric determination of hydrogen peroxide and glucose. *Microchim. Acta* **186**, (2019).

5. Choleva, T. G., Gatselou, V. A., Tsogas, G. Z. & Giokas, D. L. Intrinsic peroxidase-like activity of rhodium nanoparticles, and their application to the colorimetric determination of hydrogen peroxide and glucose. *Microchim. Acta* **185**, (2018).

6. Darabdhara, G., Boruah, P. K. & Das, M. R. Colorimetric determination of glucose in solution and via the use of a paper strip by exploiting the peroxidase and oxidase mimicking activity of bimetallic Cu-Pd nanoparticles deposited on reduced graphene oxide, graphitic carbon nitride, or MoS2 nanoshe. *Microchim. Acta* **186**, (2019).

7. Ortiz-Gómez, I. *et al.* Microfluidic paper-based device for colorimetric determination of glucose based on a metal-organic framework acting as peroxidase mimetic. *Microchim. Acta* **185**, (2018).

8. Li, F. *et al.* β-Cyclodextrin coated porous Pd@Au nanostructures with enhanced peroxidase-like activity for colorimetric and paper-based determination of glucose. *Microchim. Acta* **187**, (2020).
